# Identification of Gαi3 as a promising target for osteosarcoma treatment

**DOI:** 10.7150/ijbs.68861

**Published:** 2022-01-24

**Authors:** Zheng-jun Bian, Hua-jian Shan, Yun-Rong Zhu, Ce Shi, Min-bin Chen, Yu-min Huang, Xiao-dong Wang, Xiao-zhong Zhou, Cong Cao

**Affiliations:** 1 Department of Orthopedics, Clinical Research Center of Neurological Disease, The Second Affiliated Hospital of Soochow University, Suzhou, China.; 2 Department of Orthopedics, The Affiliated Hospital of Yangzhou University, Yangzhou University, Yangzhou, China.; 3 Department of Orthopedics, Affiliated Jiangyin Hospital of Xuzhou Medical University, Jiangyin, China.; 4 Department of Orthopedics, The Affiliated Suqian Hospital of Xuzhou Medical University, Suqian, China.; 5 Department of Radiotherapy and Oncology, Kunshan First People's Hospital Affiliated to Jiangsu University, Kunshan, China.; 6 Department of Orthopedics, The First Affiliated Hospital of Nanjing Medical University, Nanjing, China.; 7 Department of Orthopedics, Children's Hospital of Soochow University, Suzhou, China.; 8 Jiangsu Key Laboratory of Neuropsychiatric Diseases and Institute of Neuroscience, Soochow University, Suzhou, China.; 9 North District, The Affiliated Suzhou Hospital of Nanjing Medical University, Suzhou Municipal Hospital, Suzhou, China.

**Keywords:** Osteosarcoma, Gαi3, multiple receptor tyrosine kinase, Akt-mTOR, Signaling

## Abstract

Sustained activation of multiple receptor tyrosine kinases (RTKs) simultaneously is vital for tumorigenesis and progression of osteosarcoma (OS). Gαi proteins recruitment to various RTKs mediates downstream oncogenic signaling activation. The expression, functions and underlying mechanisms of Gαi3 in human OS were examined. Expression of Gαi3 is robustly elevated in human OS tissues and is correlated with a poor overall survival. In patient-derived primary OS cells and immortalized lines (MG63 and U2OS), Gαi3 depletion, by shRNA and CRISPR/Cas9 strategies, robustly suppressed cell viability, proliferation and migration, while provoking G1-S arrest and apoptosis activation. Conversely, Gαi3 overexpressing ectopically can accelerate proliferation and migration of OS cells. In OS cells, Gαi3 immunoprecipitated with VEGFR2, FGFR, PGDFR and EGFR, mediating downstream cascade transduction. Akt-mTOR activation in primary OS cells was potently inhibited by Gαi3 shRNA, knockout or dominant negative mutation, but augmented after Gαi3 overexpression. *In vivo* studies showed that Gαi3 shRNA AAV (adeno-associated viruses) intratumoral injection largely inhibited the growth of subcutaneous xenografts of primary OS cells. Moreover, the growth of the Gαi3-knockout primary OS xenografts was much slower than that of OS xenografts with empty vector. In Gαi3-depleted OS xenografts tissues, Gαi3 downregulation and Akt-mTOR inactivation were detected. Taken together, overexpressed Gαi3 mediates RTK-Akt signaling to drive OS progression.

## Introduction

For the advanced osteosarcoma (OS) patients with metastatic, recurrent or therapy-resistant OS, the prognosis is poor 1. Further exploring the novel targeted therapeutics for OS is thus extremely important 2-7. Due to various gene mutations, overexpression and/or over-activation of multiple receptor tyrosine kinases (RTKs) and its downstream oncogenic cascades are essential for initiation, progression and therapy-resistance of OS 4, 8, 9. Several RTKs, including VEGFR, PDGFR, RET, EGFR and IGFR, as well as KIT and FGFR, are key drivers for the cancerous behaviors of OS 4, 8, 9. Concurrent activation of multiple RTKs shall provoke sustained activation of oncogenic cascades, causing persistent cancer growth 8, 9. Therefore, targeting one or few RTKs, using genetic methods and/or pharmacological inhibitors, could only achieve minimal anti-OS efficiency 8, 9. The novel stragies targeting multiple RTKs simultaneously should achieve better anti-OS outcome 8, 9.

The inhibitory guanine nucleotide regulatory proteins, Gαi proteins, consists of three subunits, including Gαi1, Gαi2 and Gαi3 10. It is known that GPCRs (G protein-coupled receptors) binding to Gαi proteins and the β and γ complexes will hinder adenylate cyclase, causing cyclic AMP (cAMP) depletion 10. Such actions would be reversed by pertussis toxin 11. Few studies have explored the expression, function and potential signaling mechanism of Gαi proteins in OS. Pine *et al.,* have shown that Gαi proteins are important for the agonist-induced cAMP production in osteosarcoma cells that were derived from rat 12. Wang *et al.,* showed that pertussis toxin can inhibit bradykinin-induced Ca^2+^ mobilization in MG63 OS cells 13.

Our group has identified an essential role of Gαi proteins in transducing signals for multiple RTKs 14-20. We found that EGFR-induced Akt-mTOR activation was abolished after Gαi1/3 double knockout (DKO) or silencing 20. With VEGF stimulation, Gαi1/3 associated with VEFGR2, promoting VEGFR2 endocytosis and downstream signaling activation 16. Similarly, Gαi1/3 mediated BDNF (brain-derived neurotrophic factor)-stimulated Akt-mTOR activation 17. Therefore, by mediating signaling transduction of multiple RTKs, Gαi proteins could be important oncogenic genes and therapeutic targets for human cancer 14, 18, 21. Indeed, we have previously shown that Gαi proteins are upregulated in glioma and gastric cancers, required for cancer growth 14, 18, 21. The current study explored Gαi3 expression and potential functions in human OS.

## Methods

### Ethics

The protocols of the present study were reviewed and approved by the Ethics Committee of Soochow University and were in according to the principle of Helsinki declaration.

### Reagents

LY294002 was from Sigma Aldrich (St Louis, M.O.). The antibodies were described in our previous studies 18. All the primers, sequences, constructs and virus were synthesized by Shanghai Genechem Co. (Shanghai, China), or mentioned otherwise.

### Cells and tissues

The immortalized OS lines, MG63 and U2OS, as well as the established hFOB1.19 osteoblastic cells were cultivated as described 22-24. Patient-derived primary OS patients, namely pOS-1 and pOS-2, were described previously 22, 23. Patient-derived human osteoblasts (pOsteoblasts) were differentiated and cultured using the previously-descried protocols 25, 26. The human tissues, including the OS tumor tissue specimens and the adjacent normal bone tissue specimens 22, 23, were obtained from the written-informed consent OS patients who were all administrated at the Affiliated Hospitals of Soochow University.

### Gene detection

Protein detection by Western blotting, RNA assays by qRT-PCR and co-immunoprecipitation (co-IP) examining protein-protein interaction were extensively described early 17, 20. When necessary, lysates from the same set of the experiment were detected in the parallel gels to test different proteins. The primers were described early 16.

### Gαi3 shRNA

The lentivirus-encoded Gαi3 shRNAs and *in vitro* cell infection were described early 16. Stable cells were selected by puromycin-containing complete medium (with FBS) for additional 96h. Gαi3 silencing (with over 95-98% knockdown efficiency) in the stable cells was always verified. The control cells were infected with non-sense scramble control shRNA lentivirus (“sh-C”) 16. For the *in vivo* studies, the Gαi3 shRNA sequence or shC sequence was sub-cloned into an established adenoviral vector, adeno-associated virus 9/AAV9 construct (Genechem). Through Lipofectamine 3000 the construct was thereafter transfected to HEK-293T cells, and the shRNA-expressing AAV virus was generated and was injected to xenograft tumors.

### CRISPR/Cas9-induced Gαi3 knockout (KO)

OS cells were transfected with Cas9-expressing construct (Genechem) by Lipofectamine 3000 (Invitrogen, Shanghai, China) to establish stable cells. Next, the lenti-CRISPR/Cas-9 Gαi3 KO construct 14, 16, was transduced to Cas9-expressing OS cells, with stable cells established by using puromycin-containing medium for additional 96h. *Gαi3* KO screening was carried out and thereafter the Gαi3 KO cells were eventually established. The control OS cells were with a lenti-CRISPR/Cas-9 empty vector with non-sense small guide RNA (“Cas9-C”).

**Gαi3 overexpression and dominant negative mutation,** stable cells establishment and verification were described in our previous studies 14, 16.

### Constitutively-active mutant Akt1

OS cells were infected with the constitutively-active Akt1 (caAkt1, S473D)-expressing adenovirus (provided by Dr. Li 27, 28) for 48h. Puromycin was thereafter added for 96h to establish the stable OS cells, where expression of the caAkt1 was confirmed by Western blotting.

### Akt1/2 shRNA

The commercial available Akt1/2 shRNA lentiviral particles (sc-43609-V, Santa Cruz Biotech) were added and transfected to cultured OS cells. After 48h, cells were cultured and selected in puromycin-containing medium for another 96h. Akt1/2 silencing was always examined.

### Cellular functional studies

The cell viability detection by cell counting kit-8 (CCK-8), the EdU nuclear staining assaying of cell proliferation, propidium iodide (PI)-FACS, “Transwell” assays were carried out using the previously described protocols 18, 21, 25, 29-33.

### Apoptosis detection

Apoptosis-related assays, including the TUNEL nuclear staining, 7-AAD and Annexin V double staining, caspase-3/-9 activities measurement, and ELISA testing the cellular ssDNA (single strand DNA) contents were described early 21, 25, 31-33.

### Xenograft studies

The primary pOS1 cells (five million cells in every mouse) were subcutaneously (*s.c.*) injected to the nude mice (18.5-19.5g, half female and half male, please refer to our previous studies 22, 23). Tumor-bearing mice were then subject to the designated treatments. Tumor volumes [(length × width^2^)/2] and animal body weights were weekly recorded. Soochow University's Ethics Committee and Institutional Animal Care and Use Committee (IACUC) reviewed and approved the protocols of animal studies.

### Statistical analyses

Statistical analyses were described previously 22, 23. The numerical data in the bar graphs indicated the mean and standard deviation (S.D.). *P*-values < 0.05 were statistically significant.

## Results

### In human OS Gαi3 is upregulated

**Figure 1:**
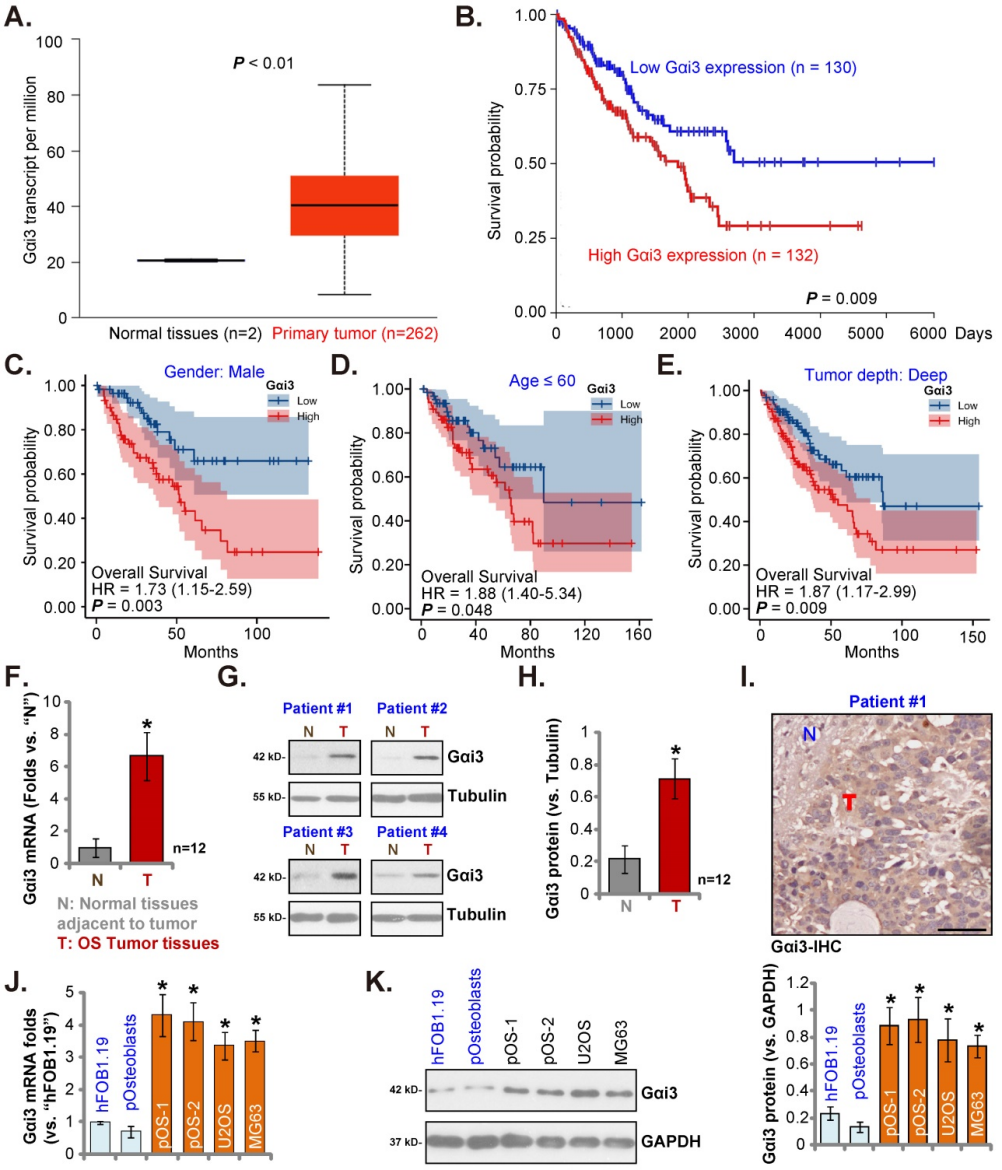
** In human OS Gαi3 is upregulated.** TCGA cohorts show *Gαi3* mRNA transcripts in 262 sarcoma cases (“Primary Tumor”) and two normal tissue cases (**A**). The Kaplan Meier Survival curve of *Gαi3*-low (n = 130, blue) and *Gαi3*-high (n = 132, red) sarcoma patients was presented (**B**). Subgroup analyses, based on the different clinical features of the OS patients, were performed as well (**C**-**E**). The expression of Gαi3 (both mRNA and protein) in twelve (n = 12) pairs of OS tumor tissue specimens (“T”) and adjacent normal bone tissue specimens (“N”) was tested (**F**-**H**). The representative human tissue Gαi3 IHC were presented as well (**I**). The expression of *Gαi3* mRNA and protein in the mentioned cells was measured (**J**-**K**). *P < 0.05 versus “N” tissues (**F** and **H**) or hFOB1.19 cells (**J** and **K**). Scale bar = 100 µm (**I**).

The Cancer Genome Atlas (TCGA) database (available on the public domain https://portal.gdc.cancer.gov) was first consulted to analyze *Gαi3* transcripts in human sarcoma tissues. Total 264 samples (HTSeq-FPKM) were collected, including the two normal specimens and the 262 sarcoma specimens. As shown, *Gαi3* transcripts in the retrieved sarcoma tissue specimens were higher than those in the retrieved normal tissue specimens (Figure **1A**). The Kaplan-Meier survival, Figure **1B**, verified that high *Gαi3* expression in sarcoma patients was correlated with a poor prognosis (HR = 1.73, ***P*** = 0.008). Subgroup analysis by different clinical features demonstrated that *Gαi3-*high expression was associated with a poor prognosis in male sarcoma patients (***P*** = 0.003, Figure **1C**), age≤60 (***P*** = 0.048, Figure **1D**), Tumor depth: deep (***P*** = 0.009, Figure **1E**).

Next, we tested Gαi3 expression in local OS tissues. As previously described 22, 23, OS tissue specimens and matched adjacent bone normal tissue specimens were retrieved from 10 different OS patients. Figure **1F** showed that *Gαi3* mRNA in the OS tissue specimens (“T”) was more than six-fold higher than that in the adjacent normal bone tissues (“N”). Gαi3 protein expression was tested as well. In the OS tissues of four representative OS patients (Patient #1/#2/#3/#4), protein expression of Gαi3 was significantly elevated (Figure **1G**). When combining all blotting results of the ten sets tissues, Gαi3 protein was found to be significantly upregulated in the OS tissues (***P*** < 0.001 versus “N” tissues, Figure **1H**). The immunohistochemistry (IHC) staining assay results, in Figure **1I**, verified Gαi3 protein elevation in OS tumor tissues (Figure **1I**), While low expression of Gαi3 was detected in the adjacent normal tissue specimens (Figure **1I**).

The expression of Gαi3 in different human OS cells was examined. Patient-derived primary OS cells (pOS-1/2, from two different OS patients), as well as the immortalized cells (MG63 and U2OS lines), were cultured. The expression of* Gαi3* mRNA in different primary and immortalized OS cells was dramatically higher than that in hFOB1.19 human osteoblastic cells and patient-derived human osteoblasts (“pOsteoblasts”) (Figure **1J**). In addition, Gαi3 protein upregulation was also shown in different primary and established OS cells (Figure **1K**). Taken together, in OS tissues and cells Gαi3 is upregulated.

### Gαi3 silencing exerts anti-tumorigenic activity in cultured OS cells

**Figure 2:**
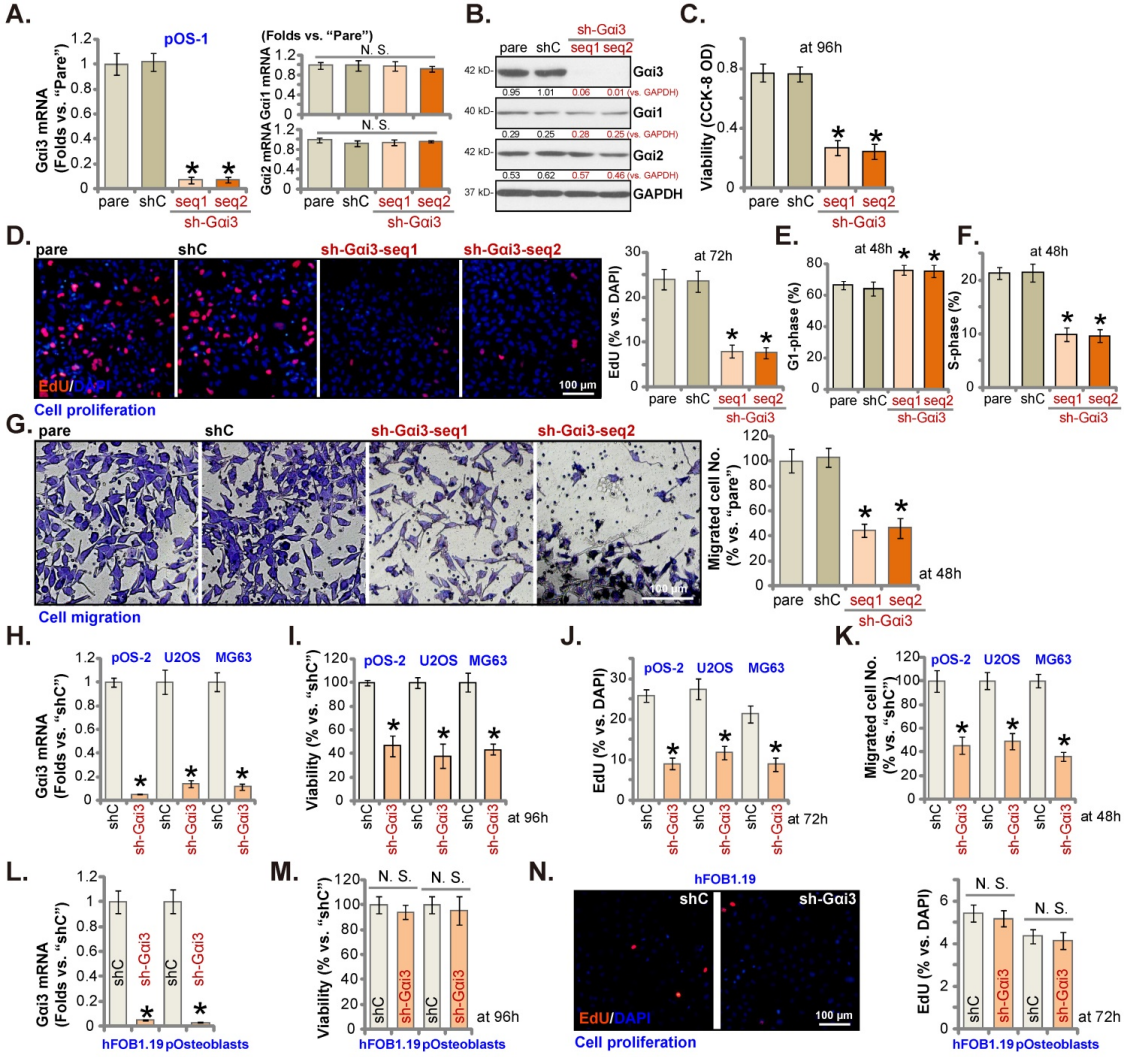
** Gαi3 silencing exerts anti-tumorigenic activity in cultured OS cells.** Patient-derived primary human OS cells (“pOS-1/2”), the immortalized OS lines (MG63 and U2OS), the immortalized hFOB1.19 cells or patient-derived primary osteoblasts (“pOsteoblasts”) were stably transduced with the designated Gαi3 lentivirus shRNA (sh-Gαi3-seq1/sh-Gαi3-seq2, with two different sequences) or the non-sense control shRNA (“shC”), the expression of indicated mRNAs and proteins were examined (**A**, **B**, **H** and **L**). After culturing for the designated hours, CCK-8 viability (**C**, **I** and **M**), and cell proliferation (testing the ratio of EdU positively stained nuclei , **D**, **J** and **N**), as well as cell cycle progression (PI-FACS assays, results quantified in **E** and **F**) and cell migration (“Transwell” assays, **G** and **K**) were tested by the listed assays. “pare” indicated the parental control OS cells. *P < 0.05 versus “shC” group. “N. S.” indicated no statistical difference (***P*** > 0.05, **A**, **M** and **N**). Each single experiment was repeated for five times. Scale bar = 100 µm (**D**, **G** and **N**).

pOS-1 primary cells were separately transfected with two different Gαi3 lentiviral shRNAs, sh-Gαi3-seq1/sh-Gαi3-seq2 22, 23. After puromycin-induced selection, Gαi3-silenced stable OS cells were established. *Gαi3* mRNA expression decreased over 90-95% in sh-Gαi3-expressing stable pOS-1 cells (Figure **2A**). Expression of *Gαi1* and *Gαi2* mRNA was unaffected (Figure **2A**). Gαi3 protein silencing was detected in the stable pOS-1 cells with Gαi3 shRNAs. Gαi1 and Gαi2 protein expression was again unchanged (Figure **2B**).

CCK-8 assays showed that Gαi3 shRNA potently decreased pOS-1 cell viability (CCK-8 OD, Figure **2C**). Moreover, Gαi3 shRNA potently inhibited pOS-1 cell proliferation, as the ratio of EdU positively stained nuclei was robustly decreased in sh-Gαi3-expressing pOS-1 cells (Figure **2D**). The PI-FACS assays were employed to test cell cycle progression. The ratio of G1-phase cells was significantly increased in pOS-1 cells expressing Gαi3 shRNA (Figure **2E**), where S-phase pOS-1 cell percentage was decreased (Figure **2F**). These results implied that the designated Gαi3 shRNAs induced G1-S arrest in primary OS cells. The “Transwell” results demonstrated that Gαi3 shRNA dramatically inhibited pOS-1 cell *in vitro* migration (Figure **2G**). The scramble control shRNA, or shC, did not significantly alter Gαi1/2/3 expression (Figure **2A**-**B**) and affect OS cellular behaviors (Figure **2C**-**G**).

Next, OS cells that were derived from another OS patient, pOS2 22, 23, and the immortalized lines (MG63 and U2OS), were stably transduced with the lentiviral Gαi3 shRNA (sh-Gαi3-seq1, “sh-Gαi3”). As shown, the designated shRNA resulted in robust Gαi3 mRNA downregulation in both primary and immortalized OS cells (Figure **2H**). Gαi3 shRNA largely suppressed CCK-8 viability (Figure **2I**), proliferation (the ratio of EdU positively stained nuclei reduction, Figure **2J**) and migration (Figure **2K**) in pOS2 primary cells and established lines.

The potential effect of Gαi3 shRNA on the Gαi3-low human osteoblasts (see Figure **1J**-**K**) was tested as well. hFOB1.19 osteoblastic cell line or patient-derived primary osteoblasts (pOsteoblasts) were stably transduced with the lentiviral sh-Gαi3-seq1, causing dramatic *Gαi3* mRNA downregulation (“sh-Gαi3”, Figure **2L**). Interestingly, in hFOB1.19 cells and pOsteoblasts, Gαi3 shRNA failed to significantly inhibit CCK-8 viability (Figure **2M**) and cell proliferation (by measuring EdU positively stained nuclei ratio, Figure **2N**), supporting a cancer cell-specific effect by Gαi3 shRNA.

### Gαi3 silencing provokes apoptosis activation in OS cells

**Figure 3:**
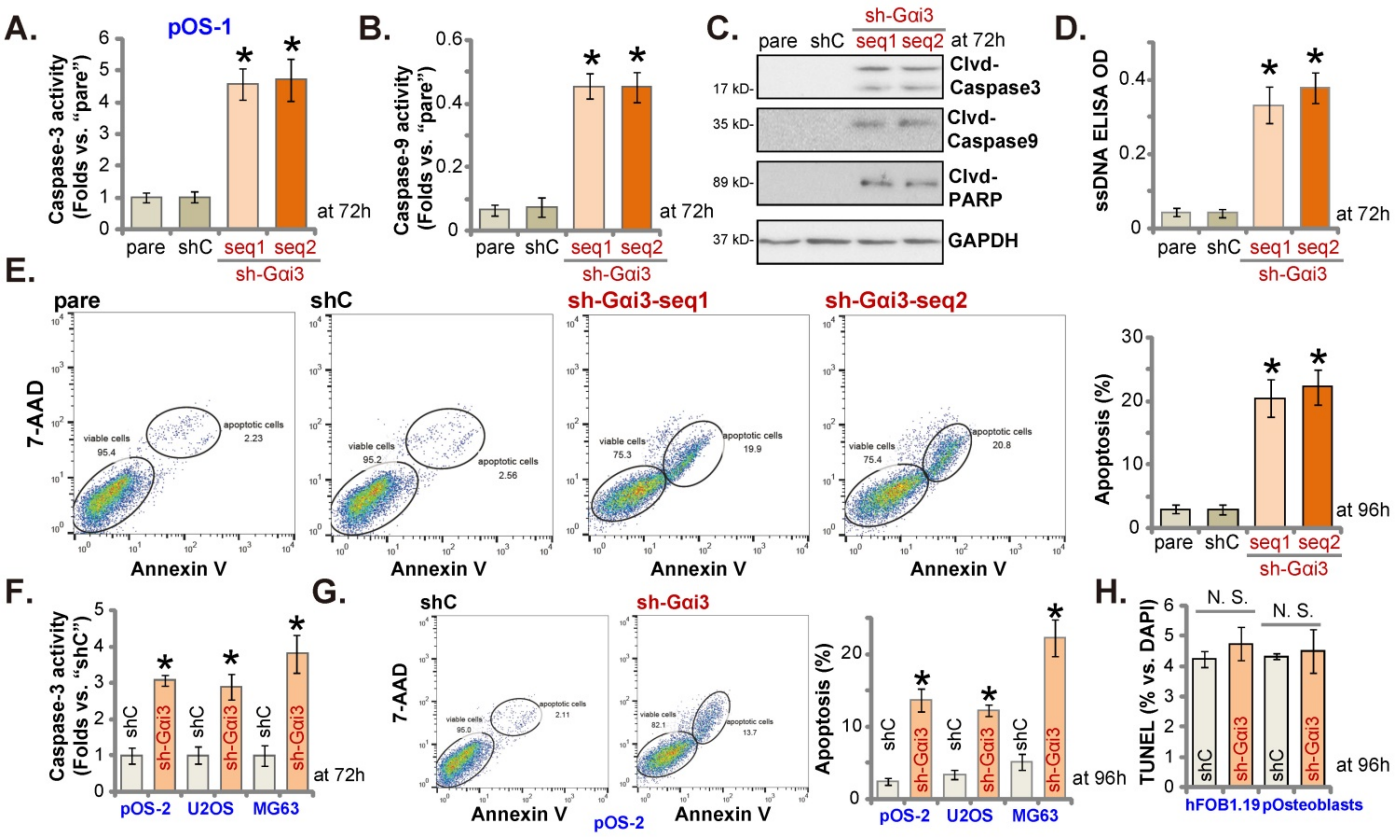
** Gαi3 silencing provokes apoptosis activation in OS cells.** Patient-derived primary human OS cells (“pOS-1/2”), the immortalized OS lines (MG63 and U2OS), the immortalized hFOB1.19 cells or patient-derived primary osteoblasts (“pOsteoblasts”) were stably transduced with the designated Gαi3 lentivirus shRNA (sh-Gαi3-seq1/sh-Gαi3-seq2, two different sequences) or the non-sense control shRNA (“shC”). After culturing for the designated hours, the caspase-PARP activation was tested (**A**-**C**, **F**), with DNA breaks tested by ssDNA ELISA assays (**D**); Cell apoptosis was examined by Annexin V-7-AAD double staining FACS (**E** and **G**) and nuclear TUNEL staining (results quantified in **H**) assays. All blotting data in this Figure were repeated five times. “pare” indicated the parental control OS cells. *P < 0.05 versus “shC” group. “N. S.” indicated no statistical difference (***P*** > 0.05, **H**). Each single experiment was repeated for five times.

Since Gαi3 silencing induced viability reduction, growth arrest, proliferation inhibition, we therefore tested its potential function on apoptosis in OS cells. Results showed that the relative activities of caspase-3 and caspase-9 (Figure **3A** and **B**) were augmented in sh-Gαi3-expressing stable pOS-1 cells. Figure **3C** showed that Gαi3 shRNA induced cleavages of caspase-3, PARP and caspase-9 in pOS-1 primary cells. Confirming increased DNA breaks in pOS-1 cells with Gαi3 shRNA, we showed that ssDNA contents were dramatically increased (tested by the ELISA assays, Figure **3D**). Moreover, FACS assay results in Figure **3E** showed that Gαi3 silencing increased the pOS-1 cell number with Annexin V-7-AAD double positive staining, confirming apoptosis activation. As expected, shC did not provoke caspase-apoptosis activation in the primary pOS-1 cells (Figure **3A**-**E**).

In pOS-2 cells and immortalized lines (MG63 and U2OS), Gαi3 shRNA (sh-Gαi3-seq1, “sh-Gαi3”, see Figure **2**) similarly augmented the relative caspase-3 activity (Figure **3F**). Apoptosis induction was observed as well in the sh-Gαi3-seq1-expressing pOS-2 cells and immortalized cell lines, evidenced by the significantly increased Annexin V-7-AAD double staining (Figure **3G**). Conversely, in the osteoblastic cell line hFOB1.19 and pOsteoblasts, shRNA-induced silencing Gαi3 (“sh-Gαi3”, see Figure **2**) failed to significantly induce apoptosis (by quantifying TUNEL-positively stained nuclei, Figure **3H**).

### Gαi3 knockout potently inhibits OS cell progression *in vitro*

**Figure 4:**
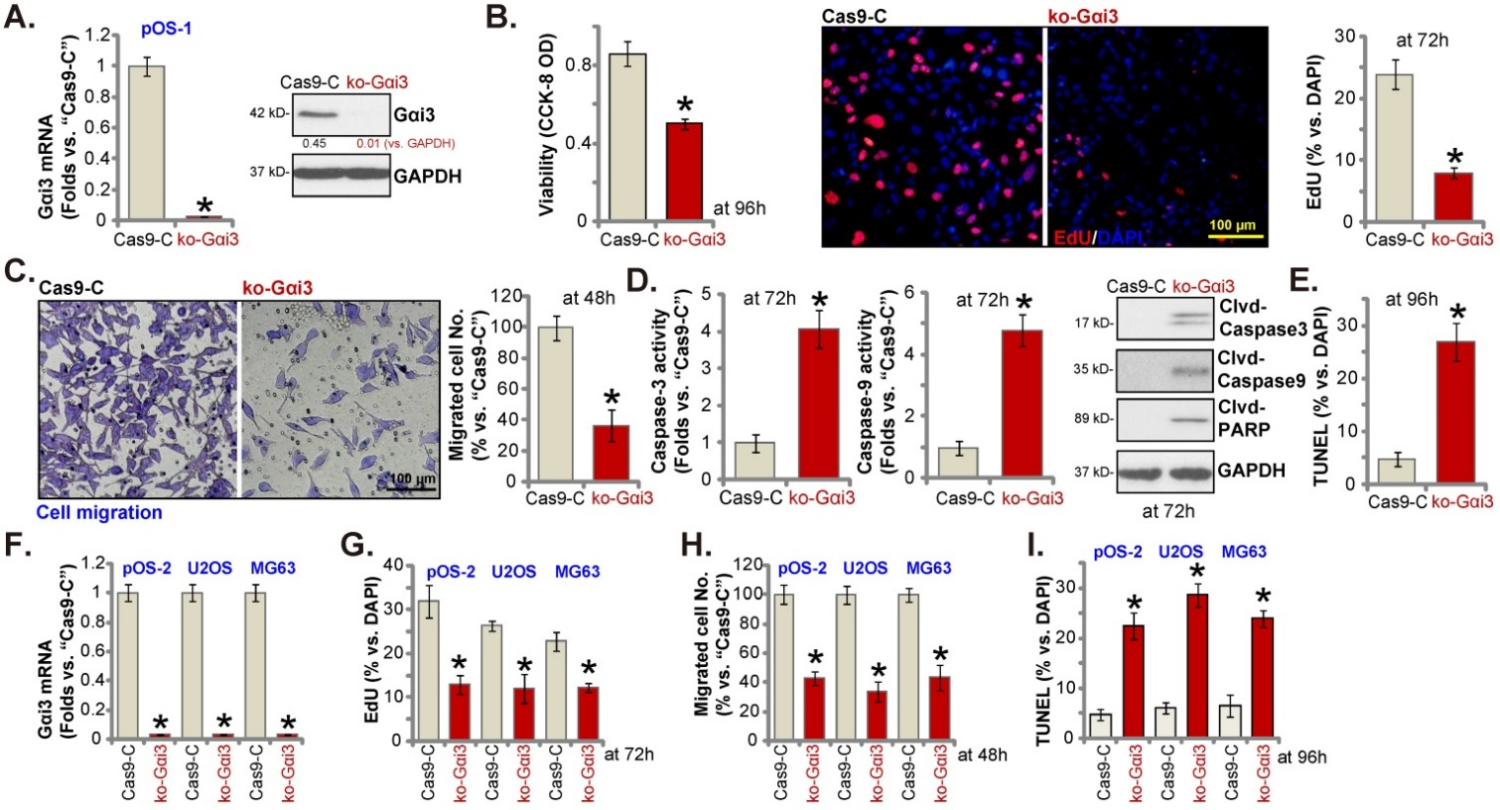
** Gαi3 knockout potently inhibits OS cell progression *in vitro*.** Patient-derived OS cells (“pOS-1/-2”) or immortalized OS lines (MG63 and U2OS), bearing the CRISPR/Cas9-Gαi3-KO-puro construct (“ko-Gαi3”) or the corresponding vector (“Cas9-C”), were established, expression of listed mRNAs and proteins were examined (**A and F**); After culturing for the designated hours, CCK-8 viability (**B**), proliferation (testing the ratio of EdU positively stained nuclei , **B and G**), cell migration (“Transwell” assays, **C and H**), caspase-PARP activation was tested (**D**), with cell apoptosis measured through quantifying the TUNEL-positively stained nuclei ratio (**E and I**). All blotting data in this Figure were repeated five times. *P < 0.05 versus “Cas9-C” group. Each single experiment was repeated for five times. Scale bar = 100 µm (**B and C**).

Next, a previously-described CRISPR/Cas9-Gαi3-KO-puro construct 15, 16 was transduced to the Cas9-expressing pOS-1 primary cells. Thereafter, single stable pOS-1 cells with the Gαi3 KO construct, or the ko-Gαi3 cells, were established after *Gαi3* KO screening, and *Gαi3* mRNA and protein (Figure **4A**) depletion was detected. Gαi3 KO largely decreased CCK-8 viability (Figure **4B**) and inhibited cell proliferation (the ratio of EdU positively stained nuclei decreasing, Figure **4B**) and migration (Figure **4C**) in pOS-1 cells. On the contrast, caspase-3 and caspase-9 activities (Figure **4D**) were augmented in ko-Gαi3 pOS-1 cells, where caspase-3, PARP and caspase-9 cleavages were induced (Figure **4D**). In addition, apoptosis induction was detected in the ko-Gαi3 pOS-1 cells, as the ratio of TUNEL-positively stained nuclei was increased significantly (see the quantified results in Figure **4E**).

The Gαi3-KO construct was employed to knockout Gαi3 in primary pOS-2 cells and immortalized lines (MG63 and U2OS), and stable cells established (“ko-Gαi3”) after screening (Figure **4F**). As shown, Gαi3 KO inhibited cell proliferation (the ratio of EdU positively stained nuclei reduction, Figure **4G**) and *in vitro* migration (Figure **4H**) in pOS-2 and established OS cells. Increased TUNEL-positive nuclei ratio confirmed apoptosis activation in ko-Gαi3 pOS-2 cells and immortalized lines (Figure **4I**). Together, Gαi3 KO, by the CRISPR/Cas9 strategy, resulted in profound anti-OS cell activity.

### Further promoting OS cell growth by Gαi3 ectopic overexpression

**Figure 5:**
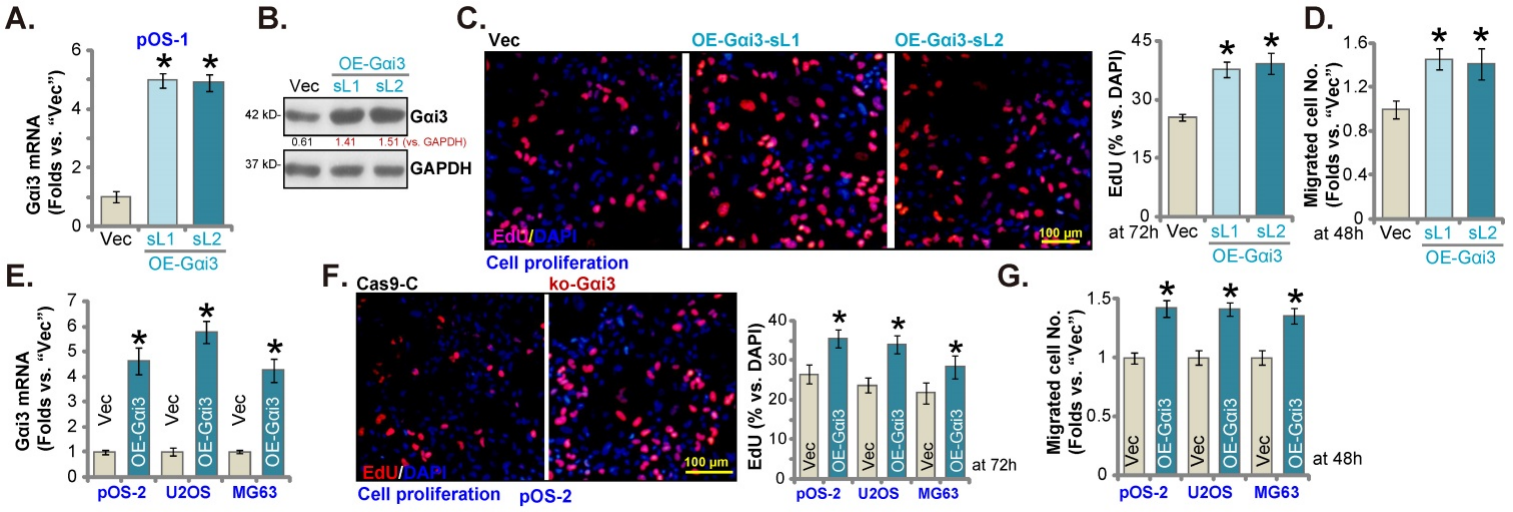
** Further promoting OS cell growth by Gαi3 ectopic overexpression.** Patient-derived primary OS cells (“pOS-1/-2”) or immortalized OS lines (MG63 and U2OS), bearing the lentiviral construct encoding wild-type Gαi3 (“OE-Gαi3”) or the corresponding vector (“Vec”), were established and cultivated, expression of listed mRNAs and proteins were measured (**A**, **B** and **E**); After culturing for the designated hours, cell proliferation (testing the ratio of EdU positively stained nuclei , **C** and **F**) and cell migration (“Transwell” assays, **D** and **G**) were measured. All blotting data in this Figure were repeated five times. *P < 0.05 versus “Vec” group. Each single experiment was repeated for five times. Scale bar = 100 µm (**C** and **F**).

Next, a Gαi3-expressing lentiviral construct (see our previous studies 14, 16) was transduced to pOS-1 cells. Following selection Gαi3-overexpressed stable pOS-1 cells were thereafter established: namely “OE-Gαi3-sL1” and “OE-Gαi3-sL2” (two selections). *Gαi3* mRNA and protein expression levels (Figure **5A** and **B**) were robustly elevated in the OE-Gαi3 pOS-1 cells. Gαi3 overexpression accelerated pOS-1 cell proliferation (the ratio of EdU positively stained nuclei increasing, Figure **5C**) and *in vitro* migration (“Transwell” assays, results quantified in Figure **5D**). In pOS-2 cells and the immortalized lines (MG63 and U2OS), ectopic overexpression of Gαi3 using the same construct (“OE-Gαi3”, Figure **5E**) enhanced cell proliferation (the ratio of EdU positively stained nuclei increasing, Figure **5F**) and *in vitro* migration (see the quantified results in Figure **5G**). Therefore, these results again supported the key cancer-promoting function of Gαi3 in OS cells.

### Gαi3 immunoprecipitates with RTKs and is key to Akt-mTOR activation in OS cells

**Figure 6:**
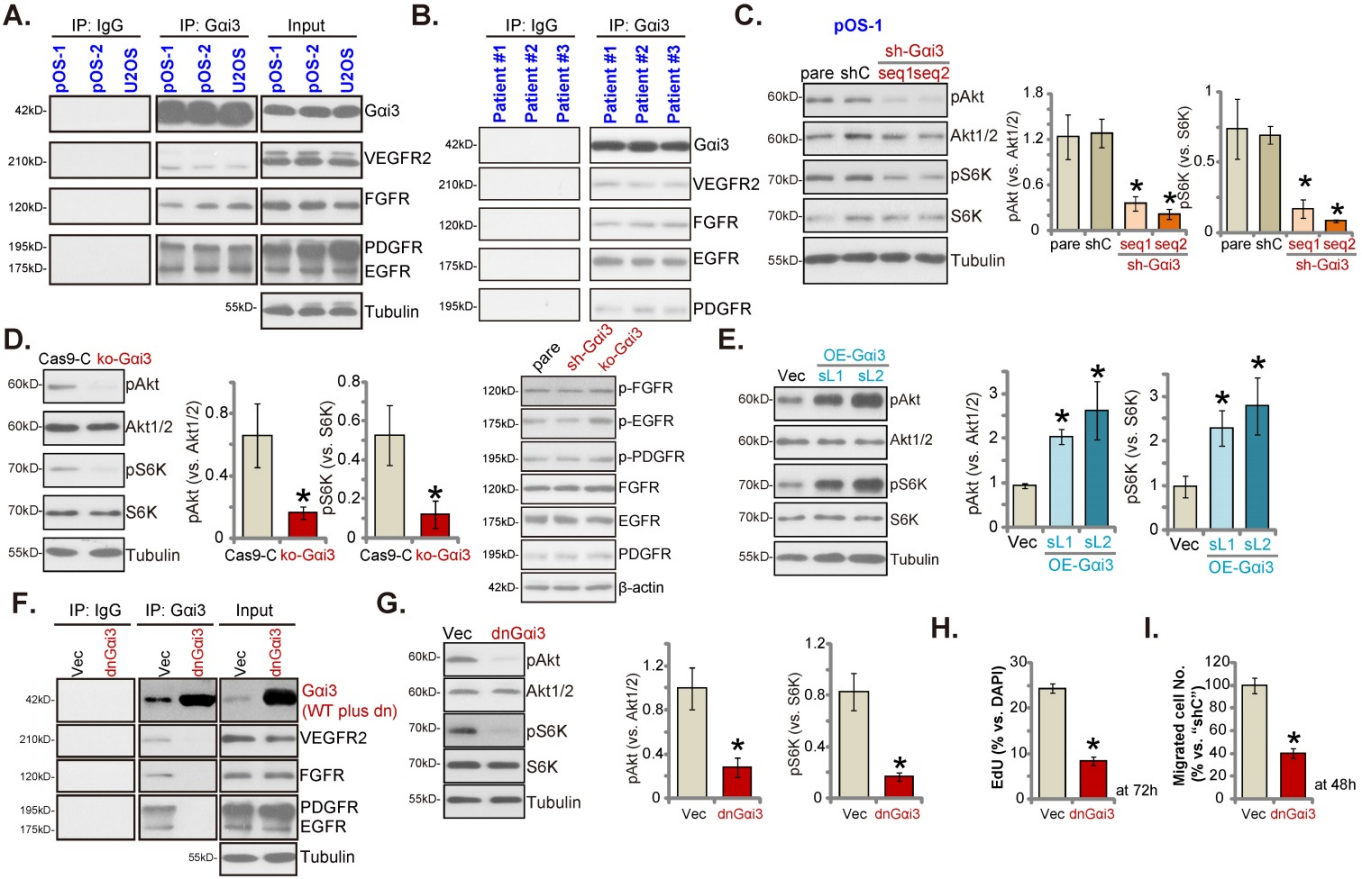
** Gαi3 immunoprecipitates with RTKs and is key to Akt-mTOR activation in OS cells.** The association between Gαi3 and the designated RTKs (VEGFR2, FGFR, PGDFR and EGFR) in patient-derived primary human OS cells (“pOS-1/2”) and U2OS line (**A**, cultured in FBS-containing medium for 5 min) as well as in OS tissues of the representative patients (**B**) was examined by the co-immunoprecipitation (Co-IP) assays. The pOS-1 primary cells stably expressing the Gαi3 shRNA (sh-Gαi3-seq1/sh-Gαi3-seq2), the CRISPR/Cas9-Gαi3-KO-puro construct (“ko-Gαi3”), the Gαi3-expressing lentiviral construct (“OE-Gαi3”), or their corresponding controls (“shC”, “Cas9-C” or “Vec”) were established, and expression of listed proteins tested (**C**-**E**). The pOS-1 primary cells, stably expressing the lentiviral dominant negative Gαi3 construct (dnGαi3) or the empty vector (“Vec”), were established, the association between Gαi3 and RTKs (VEGFR2, FGFR, PGDFR and EGFR) as well as their expression were examined (**F** and **G**). After culturing for the designated hours, cell proliferation and migration were separately examined by EdU staining (**H**) and “Transwell” (**I**) assays. All blotting data in this Figure were repeated five times. “pare” indicated the parental control OS cells. *P < 0.05 versus “shC”/“Cas9-C”/“Vec” group. Each single experiment was repeated for five times.

Our previous studies have shown that Gαi proteins associated with several oncogenic RTKs (EGFR, VEGFR2, TrkB, FGFR and KGFR), mediating downstream signaling activation 14, 16-21. Co-IP (co-immunoprecipitation) assays, Figure **6A**, demonstrated that Gαi3 immunoprecipitated with VEGFR2, FGFR, PGDFR and EGFR in primary OS cells (“pOS-1/2”) and immortalized U2OS cells. Moreover, the association between Gαi3 and multiple RTKs (VEGFR2, FGFR, PGDFR and EGFR) was detected in the human OS tissues from three representative patients (Figure **6B**). When testing downstream Akt-mTOR signaling, we showed that levels of phosphorylated-Akt (Ser-473) and phosphorylated-S6K (Thr-389) were dramatically decreased in pOS-1 cells bearing Gαi3 shRNAs (Figure **6C**). Moreover, CRISPR/Cas9-induced Gαi3 KO (see Figure **4**) largely inhibited Akt-S6K phosphorylations in pOS-1 cells (Figure **6D**). Notably, RTKs (FGFR, PGDFR and EGFR) expression and phosphorylation were unaffected by Gαi3 shRNA (sh-Gαi3-seq1) or Gαi3 KO (Figure **6D**). Conversely, ectopic overexpression of Gαi3 (see Figure **5**) significantly increased Akt-S6K activation in pOS-1 cells (Figure **6E**).

To block Gαi3-RTKs association, the lentiviral dominant negative (dn) Gαi3 mutant construct was stably transduced into pOS-1 cells. The dnGαi3 mutant will replace the conserved Gly (G) residue with the Thr (T) residue in the G3 box, thereby preventing Gαi1/3 interaction with the associated proteins 19, 20. Results show that dnGαi3 disrupted the association between Gαi3 and multiple RTKs (VEGFR2, FGFR, PGDFR and EGFR) in pOS-1 cells (Figure **6F**). Expression of RTKs was however unchanged (Figure **6F**, “Input”). Importantly, dnGαi3 largely inhibited Akt-S6K phosphorylations in pOS-1 primary cells (Figure **6G**). The dnGαi3 largely suppressed pOS-1 cell *in vitro* proliferation and migration, examined through the nuclear EdU staining (see the quantified results in Figure **6H**) and “Transwell” (Figure **6I**) assays, respectively.

### Akt-mTOR inhibition contributes to Gαi3 depletion-induced anti-OS cell activity

**Figure 7:**
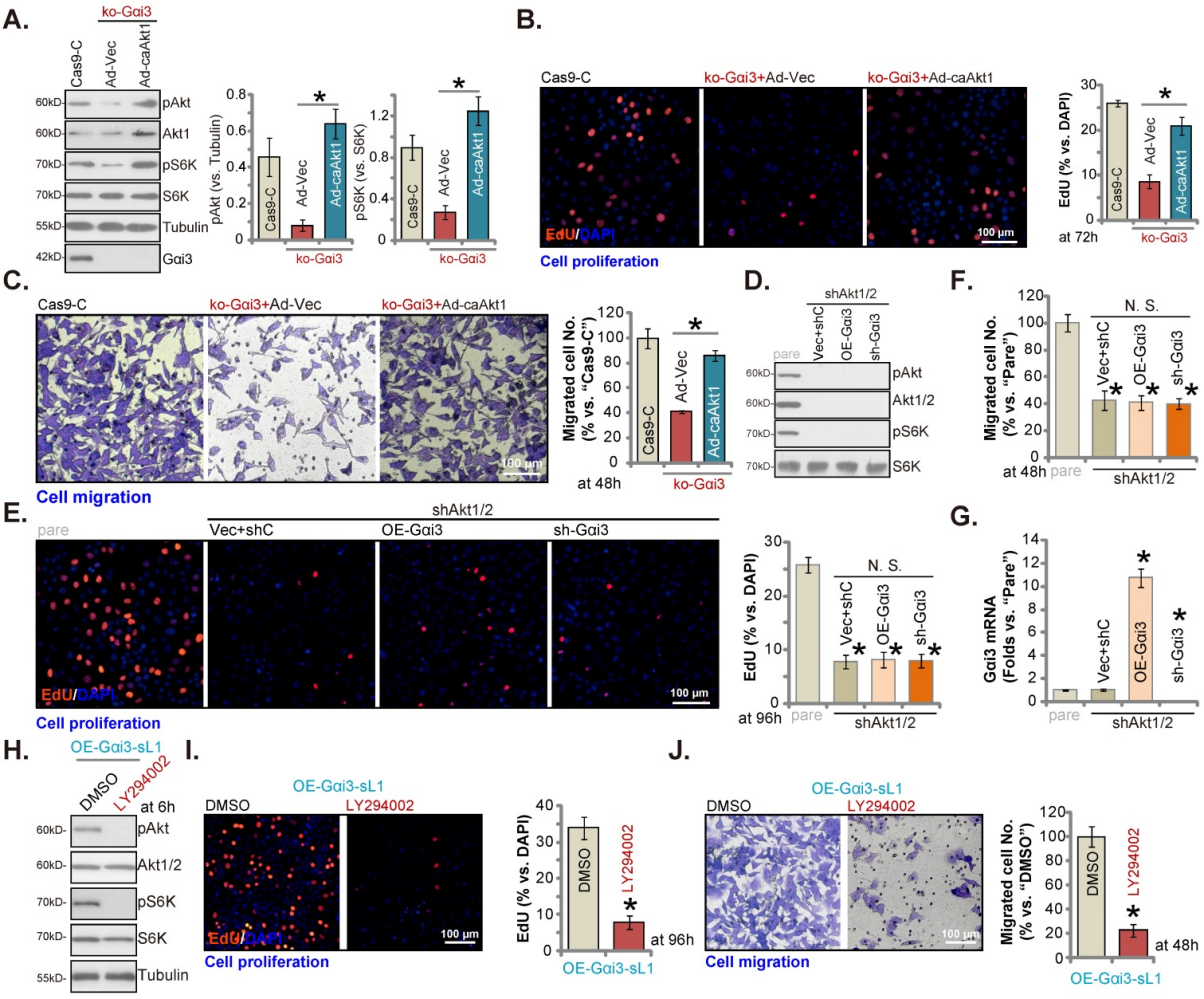
** Akt-mTOR inhibition contributes to Gαi3 depletion-induced anti-OS cell activity.** The pOS-1 cells bearing the CRISPR/Cas9-Gαi3-KO-puro construct (“ko-Gαi3”) were further infected with the constitutively-active Akt1 adenovirus (“Ad-caAkt1”) or the adenovirus with the empty vector (“Ad-Vec”), control cells were with the CRISPR/Cas9 empty vector (“Cas9-C”), listed proteins were shown (**A**). Cells were cultured for designated hours, cell proliferation (**B**, EdU assays) and migration (**C**, “Transwell” assays) were tested. pOS-1 cells stably bearing the lentiviral Akt1/2 shRNA (“shAkt1/2”) were further transduced with a wild-type Gαi3 (“OE-Gαi3”) lentiviral construct, the lentiviral sh-Gαi3-seq1 (“sh-Gαi3”) or their control construct (“Vec+shC”), stable cells were established. *Gαi3* mRNA and listed proteins were shown (**D** and **G**). After culturing for the designated hours, cell proliferation (**E**, by measuring EdU positively stained nuclei ratio) and migration (**F**) were measured. pOS-1 cells, bearing the lentiviral construct encoding wild-type Gαi3 (“OE-Gαi3-sL1”) were treated with LY294002 (150 nM) or the vehicle control (0.1% DMSO), and cultured for designated time periods, listed proteins were shown (**H**), with cell proliferation (**I**) and migration (**J**) examined as well. All blotting data in this Figure were repeated five times. “pare” indicated the parental control OS cells. *P < 0.05 (**A**-**C**). *P < 0.05 versus “pare” cells (**E**-**G**). *P < 0.05 versus “DMSO” (**I** and **J**). “N. S.” indicated no statistical difference (***P*** > 0.05, **E** and **F**). Each single experiment was repeated for five times. Scale bar = 100 µm (**B**, **C**, **E**, **I** and **J**).

To support that Akt-mTOR inhibition was the main mechanism of Gαi3 depletion-caused anti-OS cell activity, we expressed the constitutively active Akt1 (caAkt1) 34 adenovirus (“Ad-caAkt1”) that could rescue Akt and S6K phosphorylation in koGαi3 pOS-1 cells (Figure **7A**). Significantly, Ad-caAkt1 restored proliferation (by quantifying EdU-positively stained nuclei, Figure **7B**) and *in vitro* migration (“Transwell” assays, Figure **7C**) of koGαi3 pOS-1 cells. Furthermore, shRNA-induced silencing of Akt1/2 (Figure **7D**) blocked Akt-S6K phosphorylations (Figure **7D**) and mimicked Gαi3 depletion-induced actions, suppressing pOS-1 cell proliferation (Figure **7E**) and *in vitro* migration (Figure **7F**). Significantly, re-introducing the Gαi3 shRNA lentivirus or the Gαi3-expresing construct (Figure **7G**) was unable to further influence cell proliferation (Figure **7E**) and migration (see quantified results in Figure **7F**) in Akt1/2-silenced pOS-1 cells. Moreover, in the Gαi3-overexpressed pOS-1 cells (OE-Gαi3-sL1), treatment with LY294002, a PI3K-Akt inhibitor 35, blocked Akt-S6K phosphorylation (Figure **7H**) and inhibited cell proliferation (Figure **7I**) and *in vitro* cell migration (Figure **7J**). Thus, Akt-mTOR inhibition should be responsible for Gαi3 depletion-induced anti-OS cell activity.

### Gαi3 depletion inhibits OS cell growth *in vivo*

**Figure 8:**
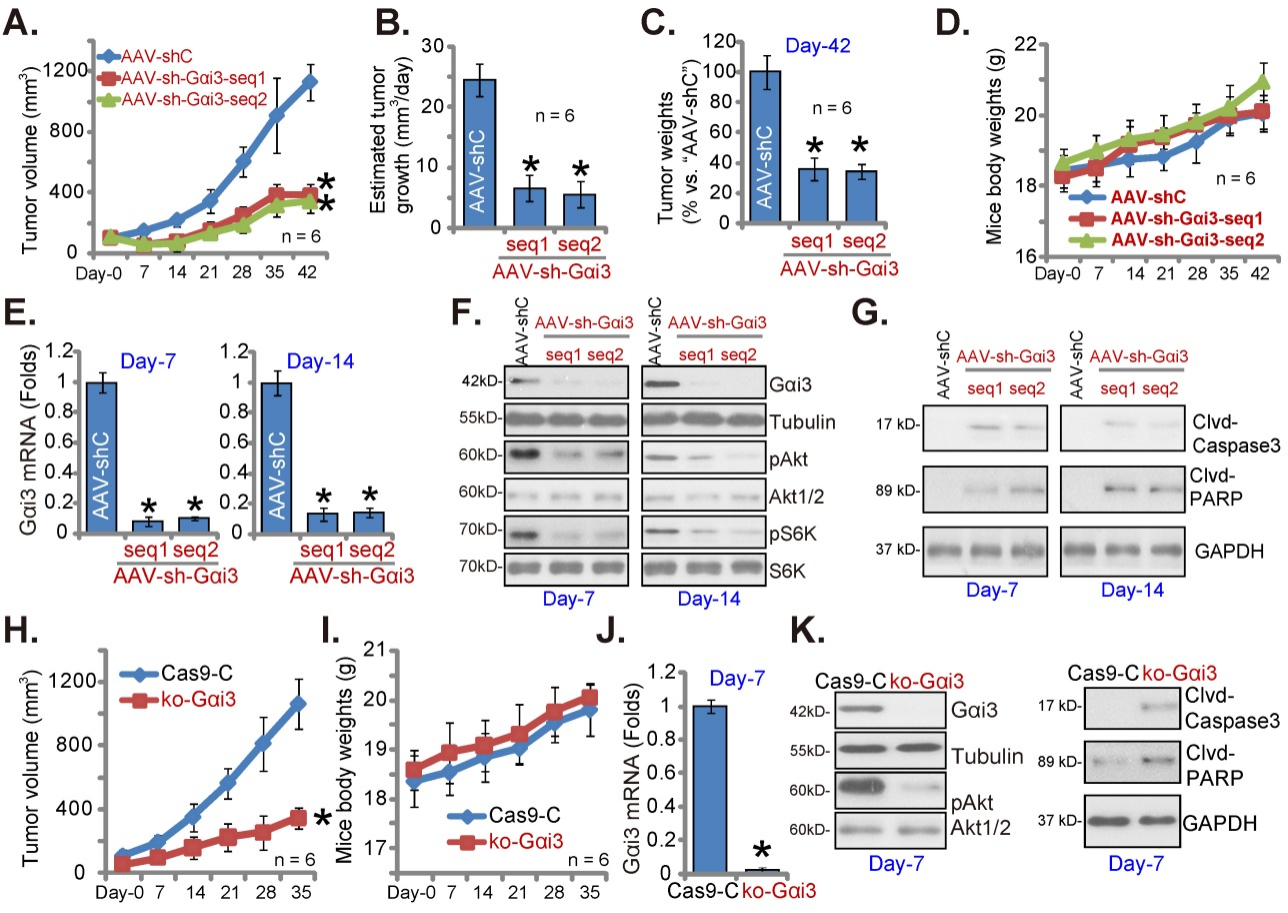
** Gαi3 depletion inhibits OS cell growth *in vivo***. The pOS-1 xenografts-bearing nude mice were intratumorally injected (daily) with the AAV-packed Gαi3 shRNAs (AAV-sh-Gαi3-seq1 or AAV-sh-Gαi3-seq2, two different sequences) or AAV-packed control shRNA (AAV-shC) for 12 days. The mice images was shown (**A**); The tumor volumes (**A**) and the mice body weights (**D**) were recorded weekly (“Day-0” to “Day-42”, total 42 days). The estimated daily tumor growth was calculated and presented (**B**). At Day-42, all pOS-1 xenograft tumors were isolated and weighted (**C**). Expression of listed genes and proteins in indicated tumor tissues lysates was shown (**E**-**G**) assays. The ko-Gαi3 pOS-1 cells and Ca9-C control cells were *s.c.* injected to the nude mice. After 20 days, tumor volumes recordings were started (“Day-0”). Weekly tumor volumes (**H**) and the mice body weights (**I**) were presented. Expression of listed genes and proteins in the described tumor lysates was tested (**J** and **K**). All blotting data in this Figure were repeated five times. *P < 0.05 versus “AAV-shC”/“Ca9-C” group.

At last, pOS-1 cells were subcutaneously (*s.c.*) injected to the nude mice. Within 20 days of cell inoculation, the subcutaneous pOS-1 xenograft tumors were established and each tumor volume was close to 100 mm^3^ (“Day-0”). The xenograft-bearing mice were thereafter assigned into three different groups randomly, with six mice in every group (n = 6). Afterwards, the mice were intratumorally injected daily with the AAV-packed Gαi3 shRNAs (AAV-sh-Gαi3-seq1 or AAV-sh-Gαi3-seq2, two different sequences in the AAV9 construct) or AAV-packed control shRNA (AAV-shC), for 12 consecutive days. Figure **8A**, recording tumor growth, demonstrated that the growth of pOS-1 xenograft tumors was robustly mitigated after AAV-sh-Gαi3 injection. The volumes of AAV-sh-Gαi3-injected pOS-1 xenografts were dramatically lower than those of AAV-shC-injected pOS-1 xenografts (Figure **8A**). The estimated daily tumor growth (see previous studies 22, 23) results demonstrated that subcutaneous pOS-1 xenograft growth was dramatically suppressed with AAV-sh-Gαi3 injection in the nude mice (Figure **8B**). At Day-42, all the animals were anaesthetized and decapitated, and pOS-1 xenografts carefully isolated and weighted. The pOS-1 tumors with AAV-sh-Gαi3 injection were significantly lighter than pOS-1 tumors with the control shRNA virus injection (Figure **8C**). Among the three mice groups there was no significant difference in the mice body weights (Figure **8D**).

At experimental Day-7 and Day-14, 3h after virus injection, one tumor of each group was carefully isolated, and total six tumors were obtained. *Gαi3* mRNA was dramatically decreased in AAV-sh-Gαi3-injected pOS-1 xenograft tissues (Figure **8E**), where Gαi3 protein downregulation as well as p-Akt and p-S6K inhibition were detected (Figure **8F**). Supporting apoptosis activation, we showed that cleaved-caspsae-3/cleaved-PARP levels were augmented in Gαi3-silenced pOS-1 xenograft tissues (Figure **8G**). Thus, intratumoral injection of AAV-packed Gαi3 shRNA suppressed Akt-mTOR activation and provoked apoptosis in pOS-1 xenografts.

In addition the ko-Gαi3 pOS-1 cells and the Ca9-C control cells (see Figure **4**) were injected to the nude mice, forming subcutaneous xenografts. After 20 days, tumor recordings were started (“Day-0”). As shown ko-Gαi3 pOS-1 xenograft growth was slower than the Ca9-C xenografts (Figure **8H**), while animal body weights were indifferent (Figure **8I**). At experimental Day-7, we carefully isolated one tumor xenograft per group. *Gαi3* mRNA and protein (Figure **8J-K**) expression was completely depleted in ko-Gαi3 pOS-1 xenograft tissues, where p-Akt was decreased (Figure **8K**). The cleaved-caspsae-3 and the cleaved-PARP levels were increased in Gαi3-KO xenograft tissues (Figure **8K**), supporting apoptosis induction *in vivo*.

## Discussion

The GPCR superfamily is composed of the immense structural and functional different proteins, participating in various biological processes and signals in the bone 36. Due to gene mutations, depletion or overexpression, GPCR components are dysregulated in human OS 36. Their roles in OS progression have been established 36. For example, Iyer *et al.,* found that A3 adenosine receptor (A3AR) depletion activated protein kinase A (PKA)-Akt-nuclear factor (NF)-κB signaling to promote OS cell growth 37. High GPR56 (G protein-coupled receptor 56) expression is an unfavorable prognostic factor, promoting invasion and proliferation of OS cells 38. Liu *et al.* demonstrated that GPR110 (G protein-coupled receptor 110) silencing inhibited OS cell growth 39.

Importantly, studies have reported that Gαi-coupled GPCRs, including Apelin receptors 40, CXCR4 41-43, melatonin receptors 44, are important contributor for OS progression. Due to various gene mutations (or overexpression), concurrent and sustained activation of multiple different RTKs in OS will provoke sustained activation of oncogenic signaling, leading to persistent OS growth and progression 4, 8, 9. Interestingly, we have previously shown that Gαi proteins are essential for signalings by several important oncogenic RTKs (including EGFR 20, VEGFR2 16, KGFR 19, FGFR 18 and TrkB 17) as well as the non-RTK receptor (IL-4R 15).

After showing the essential role of Gαi 1/3 in activation of oncogenic signalings by RTKs, we previously explored Gαi 1/3 in different human cancers. Gαi 1 and Gαi 3 expression is elevated in glioma, correlating with tumor stage 14, 18. Gαi1 can form a complex with multiple RTKs (including FGFR, PDGFR and EGFR), transducing downstream Akt-mTOR activation in glioma tissues and cells 18. Conversely, Gαi1 silencing or mutation inhibited glioma cell growth 18. In the mouse brain, the orthotopic growth of patient-derived glioma xenografts was arrested after Gαi 1/3 depletion, whereas forced overexpression of Gαi 1/3 enhanced growth 14. We also showed that Gαi 1 upregulation in human gastric cancer was correlated with poor overall survival 21. Gαi 1 silencing or knockout inhibited Akt-mTOR activation and gastric cancer cell growth 21. These previous studies supported that Gαi 1/3 could be important oncogenic genes and promising therapeutic targets of human cancer.

Gαi3 should be a vital gene for OS progression. TCGA database shows that transcripts of *Gαi3* are significantly upregulated in sarcoma tissues, and high-*Gαi3* expression in sarcoma correlating with the poor overall survival. Gαi3 elevation was observed in local OS tissues as well as in different immortalized and primary OS cells, while low expression was observed in cancer-surrounding normal bone tissues and in immortalized and primary osteoblasts. Functional studies showed that in different OS cells, Gαi3 depletion, by shRNA or CRISPR/Cas9 strategies, robustly suppressed cell survival, proliferation and cell migration, and provoking G1-S arrest and apoptosis. Contrarily, ectopic Gαi3 overexpression can further accelerate OS cell growth.* In vivo*, Gαi3 shRNA AAV intratumoral injection potently suppressed the growth of the patient-derived OS xenografts in nude mice. Moreover, the growth of primary OS xenografts of the Gαi3 KO cells was largely suppressed.

We have previously discovered that Gαi1/3 association with multiple RTKs was required for downstream signaling activation. For instance, Gαi1/3 are key proteins in mediating VEGF-induced VEGFR2 signaling 16. Following VEGF stimulation, Gαi1/3 were in the VEGFR2 endocytosis complex, required for VEGFR2 endocytosis and subsequent activation of downstream signalings 16. Similarly, Gαi1/3 proteins are indispensable signaling molecule for EGF- and KGF-induced Akt-mTORC1 signaling activation 19, 20. In addition, brain-derived neurotrophic factor (BDNF)-induced signaling and anti-depressive actions required Gαi1/3 17. Gαi 1/3 silencing inhibited BDNF-induced TrkB endocytosis and activation of the downstream signaling 17.

The present study implied that Gαi3-driven OS cell growth was primarily through mediating Akt-mTOR cascade activation. In OS cell and tissues Gαi3 associated with RTKs (VEGFR2, FGFR, PGDFR and EGFR), essential for downstream Akt-mTOR activation. In OS cells Akt-S6K activation was robustly suppressed by Gαi3 shRNA/KO, but augmented after Gαi3 overexpression. *In vivo*, Akt-S6K phosphorylations were decreased in Gαi3-depleted OS xenografts tissues. Importantly, disrupting Gαi3-RTKs association, through dnGαi3, largely inhibited Akt-S6K activation and OS cell proliferation and migration. Restoring Akt-S6K activation, by caAkt1, rescued proliferation and migration of Gαi3-KO OS cells. Conversely, mimicking Gαi3 depletion-induced actions, Akt1/2 silencing inhibited OS cell proliferation and migration. Significantly, exogenously altering Gαi3 expression failed to affect proliferation and migration in Akt1/2-silenced cells. Therefore, Gαi3-driven OS cell growth was possibly due to mediating RTKs-Akt signaling.

## Conclusion

Over three-fifths of bone sarcoma are OS 45, 46. The standard chemotherapy of OS in clinic is the combination of methotrexate, doxorubicin, and cisplatin 45, 47, 48, showing limited success in metastatic and other OS patients with advanced diseases 45, 47, 48. Further exploring key pathologic mechanisms and the driving signaling molecule for advanced OS is therefore important 45, 47, 48. The results of this study showed that overexpressed Gαi3 mediated RTKs signaling to drive OS progression, serving as a novel and promising treatment molecular target for patients with OS.
